# The mechanical properties of individual cell spheroids

**DOI:** 10.1038/s41598-017-07813-5

**Published:** 2017-08-04

**Authors:** Alice Blumlein, Noel Williams, Jennifer J. McManus

**Affiliations:** Department of Chemistry, Maynooth University, Maynooth, Co. Kildare, Ireland

## Abstract

The overall physical properties of tissues emerge in a complex manner from the properties of the component cells and other constituent materials from which the tissue is formed, across multiple length scales ranging from nanometres to millimetres. Recent studies have suggested that interfacial tension between cells contributes significantly to the mechanical properties of tissues and that the overall surface tension is determined by the ratio of adhesion tension to cortical tension. Using cavitation rheology (CR), we have measured the interfacial properties and the elastic modulus of spheroids formed from HEK cells. By comparing the work of bubble formation with deformation of the cell spheroid at different length scales, we have estimated the cortical tension for HEK cells. This innovative approach to understanding the fundamental physical properties associated with tissue mechanics may guide new approaches for the generation of materials to replace or regenerate damaged or diseased tissues.

## Introduction

To form healthy mature tissues and organisms, cells must grow, differentiate and self-organise in both a spatial and temporal manner. These processes are regulated by signalling molecules and changes in cell adhesion, interfacial tension and the mechanical properties of the microenvironment^1, 2^. Failure to regulate these control mechanisms can result in the formation of tumours and other diseased states including Alzheimer’s disease and kidney dysplasia^3, 4^. Furthermore, biomechanics strongly influence the processes involved in tissue degeneration and repair^5, 6^. Therefore, it is important to understand the physical factors involved in the development of healthy tissue since strategies to replace or regenerate injured or diseased tissue are limited by an incomplete understanding of the mechanical properties of normal tissues^7^.

The mechanical properties of tissues, that is, the mechanical response of the tissue averaged over a large number of cells, emerge in a complex manner from the properties of individual components that make up the system and the interplay between these components, across multiple length scales^8^. The biophysical properties of the cytoskeleton and cell membrane determine the mechanical properties of individual cells in isolation, while the material properties of multicellular aggregates and tissues arise through complex associations of cell adhesion molecules with each other, the cytoskeleton and the extracellular matrix^7, 9^. Kuo and co-workers recently conducted experiments on embryonic chick tendons and demonstrated that the elasticity of the tendons depended upon different tissue and cellular components at different developmental stages^9^. Using atomic force microscopy, they demonstrated that crosslinking of collagen fibers in the extracellular matrix contributes significantly to the elastic modulus during late embryonic stages, however, this is not the case in the early embryonic stages where a well-organised actin cytoskeleton increases the elastic modulus^7, 9^.

Recent advances in embryogenesis suggest that the interfacial tension between cells provides a significant contribution to the overall mechanical properties of cellular materials^2, 10–12^. Two theories regarding the origin of tissue surface tension have been proposed, the differential adhesion hypothesis (DAH)^10, 13, 14^ and more recently, the differential interfacial tension hypothesis (DITH)^2, 15, 16^. Whereas, DAH considers only the relationship between adhesive energy and surface tension, DITH also recognises a contribution from cortical tension (tension conferred by a layer of actin beneath the cell membrane) and that it is the ratio of adhesion tension to cortical tension that determines the overall surface tension. The cortical tensions of individual cells have been reported to range from 10^−3^–10^−5 ^mNm^−1^ ^17–21^, while tissue interfacial tensions have been reported from 1.6 to 20 mNm^−1^ ^2, 22–24^. Measurements of the elastic modulus for a variety of cell types have also been determined, using methods such as such as Atomic Force Microscopy (AFM) and Micropipette Aspiration (Table 1 and references therein) and other more recent methods^25, 26^. For epithelial cells, elastic modulus values between 150 and 10,000 Pa have been measured, depending on the method used, the cell type and the region of the cell probed (whole cell, cytoplasm or nucleus) and the experimental conditions (see Table 1).

**Table 1:** Elastic modulus measurements for different cell types.

Cell type	Method	Elastic Modulus (*Pa*)	Reference
Human aortic endothelial cell	AFM	1,000–5,000	27
Porcine/murine cerebral capillary endothelial cells	AFM	5,000	28
Human umbilical vein endothelial cell	AFM	5,000–10,000	29
Human umbilical vein endothelial cell	AFM	1,400 (near edge)	30
		6,800 (nucleus)	
		3,000 (in between)	
Schlemm’s canal endothelial cells	AFM	1,000–3000	31
Breast epithelial cancer cells	AFM	500–2,000	32
Rat hippocampus	AFM	201	33
Human umbilical vein endothelial cell	Magnetic twisting cytometry	400	34
Bovine thoracic aortic endothelial cell	Micropipette aspiration	400 (nucleus)	35
Oocyte –zona pelludica	Micropipette aspiration	7470	36
Human mesenchymal stem cells	Micropipette aspiration	372	37
Chondrocytes	Micropipette aspiration	500	18
Murine sarcoma aggregate	Micropipette aspiration	700	23
Colon cancer cell lines	Micropipette aspiration	56–81	38
Bovine aortic endothelial cell	Compression between microplates	300–700 (cytoplasm)	39
		4,000–8,000 (nucleus)	
Chondrogenic progenitor cells	Micropipette aspiration	180–350	40

Understanding the role of interfacial, cortical and adhesion tension during embryogenesis is the first step towards tuning these interactions and opens the door to the directed self-organisation of cells in artificial tissues^41, 42^. Changes in the expression of adhesion proteins and the resulting change in cortical and interfacial tension have been associated with the progression of cancer and metastasis^43–46^. Thus, the ability to measure cortical and interfacial tension and adhesive energy of cells within tissues could lead to advances in several different fields.

Cavitation rheology can be used to determine both the interfacial tension and the modulus of biological materials^47–49^. By varying the needle diameter, these mechanical properties may be measured at intracellular, cellular and intercellular length-scales. Cell spheroids are an ideal system in which to measure the mechanical properties of cellular materials across these length scales. Unlike whole tissues, when a single cell type is used to form the spheroid, compositionally identical replicates can easily be grown. Furthermore, unlike explants (excised tissue), other factors including age and the biochemical environment, which have been shown to alter the mechanical characteristics of cells and tissues, can be rigorously controlled^50–52^.

We have measured the deformation of cell spheroids using cavitation rheology at length scales ranging from 5 μm to 30 μm. In interpreting the data, we have accounted both for the size of the cell spheroid and any 1D confinement effects that may arise due to the sample environment in interpreting the critical pressure. By calculating the energy associated with the bubble formation and comparing this with the binding energies associated with cell surface proteins, we have estimated the cortical tension for HEK cells which to the best of our knowledge has not previously been reported. These data further our understanding of the fundamental biophysical phenomena associated with the formation of tissues and thus may contribute to the advancement of strategies to replace or regenerate injured or damaged tissues.

## Results and Discussion

**Figure 1:**
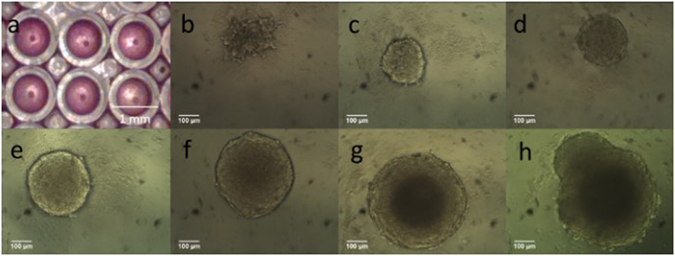
HEK 293T/17 spheroids grown using the liquid overlay method (1% agarose). Growth was monitored over eight days. (**a**) overview of cells in a section of a 96 well plate, scale bar = 1 mm; (**b**–**h**) day 2–day 8, scale bar = 100 μm. A grid, of known dimensions, was used to convert length in pixels to length in mm (3,157 pixels = 1 mm using a 10x objective).

The growth of HEK cell spheroids was monitored by light microscopy over several days, Fig. 1. After 24 hours, the cells formed loose, asymmetric clusters. By day 2 more defined spheroids with an average diameter of 209 ± 10 μm (n = 60) were observed, Fig. 1.

**Figure 2:**
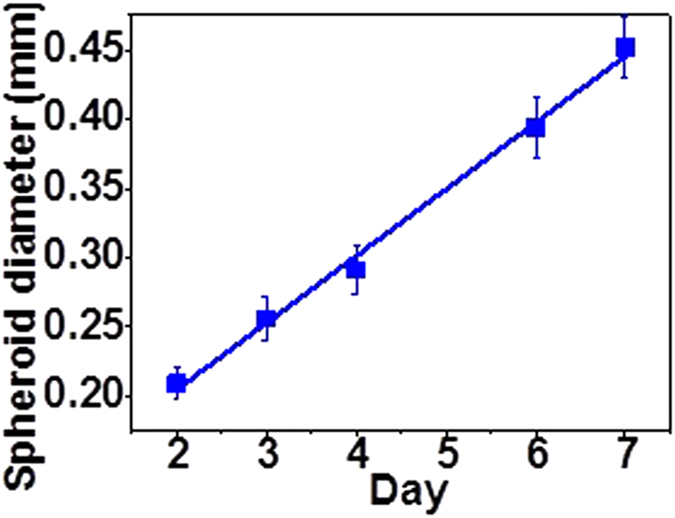
Growth curve for HEK 293T/17 spheroids grown using a liquid overlay method (1% agarose) seeded at 1.5 × 10^3^ cells/ well; 60 replicates per point. Growth rate ~50 μm/day.

Due to the limited diffusion of oxygen, in most tissues cells are located no further than 100 to 200 μm from the nearest capillary^53^, similarly, unless positioned within 100 to 200 μm from the nearest capillary, the cells of laboratory grown implants do not survive^54^. In spheroids, a necrotic core develops, due to hypoxia^55^, when the spheroid grows too large and the interior cells are located beyond the diffusion limit of approximately 200 μm from the rim of the spheroid^56, 57^. At day 8, 36% of the spheroids developed outgrowths (Fig. 1), consistent with a breach of the diffusion limit and possible ejection of the necrotic core^58^. Therefore, the spheroids were harvested on day seven when the spheroids were sufficiently large for cavitation rheology measurements (Fig. 2), but before any evidence of outgrowths due to the presence of a neurotic core might be present^59^.

To a first approximation in cavitation rheology (CR)﻿, the pressure-growth relationship for a spherical void in a viscoelastic material is;

1 $${P}_{c}=\frac{5}{6}E+\frac{2\gamma }{r}$$

where *P*_*c*_ is the critical pressure, the maximum inflation pressure^47, 60^, *E* is the modulus, *γ* is the surface tension and *r* is the needle radius (inner part). Equation 1 is valid for the “thickshell” case, where the sample volume is large relative to the size of the expanding cavity, i.e. when the outer boundary of the material is sufficiently far from the cavity origin to remain unperturbed by the cavity formation^61^. When the sample volume decreases the radius of the deformation (which scales with the needle radius) may begin to approach the “thickshell” limit. Spheroids are small (<1 mm) and the bubble growth may begin to approach the outer boundary of the spheroid and exceed the “thickshell” limit required for CR. A modified relationship between the critical pressure and the elastic modulus to account for this situation was reported by Solomon and co-workers^62^.

2 $${P}_{c}=\frac{5}{6}E[\begin{array}{c}\frac{6a}{5}{(\begin{array}{c}\frac{{R}_{i}+H}{{R}_{i}}\end{array})}^{b}+1\end{array}]+\frac{2\gamma }{r}$$

where *a* and *b* are fitting parameters, equal to −0.86 and −0.65, respectively﻿﻿, *﻿﻿R*_i_ is the needle diameter and *H* is the thickness of the elastic shell^63^. The mechanical properties of the spheroids need to be measured while the cells are bathed in cell culture medium. This adds an extra level of complexity, since these small objects can float away from the needle during insertion. It is therefore necessary to gently “trap” the spheroid against the side of the container to prevent this movement. If the growth of the bubble within a trapped spheroid is beyond the “thickshell” limit, then a 1-D confinement effect may also contribute to the measurement of a critical pressure in the spheroid. However, this 1D confinement will not materialise without a breach of the “thickshell” limit.

**Figure 3:**
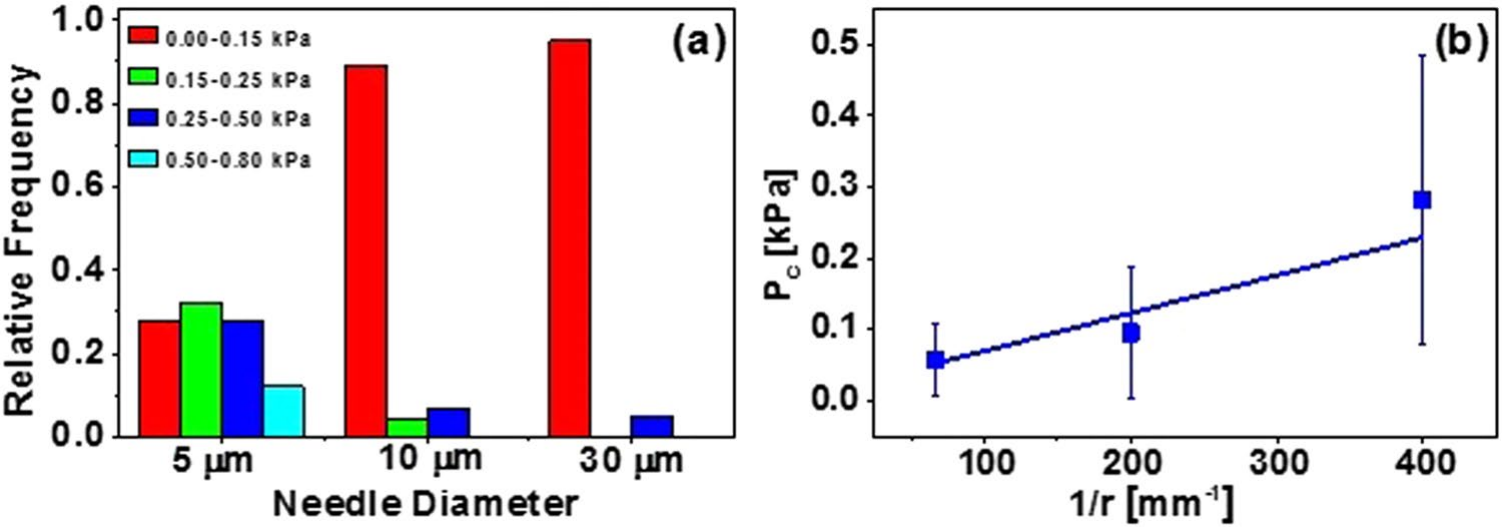
**(a)** Range of critical pressure values for HEK 293 T/17 spheroids on day 7 and the relative frequency of critical pressures. **(b)** The elastic modulus and interfacial tension (γ) of HEK/17 spheroids on day 7. E ≈ 20 Pa, γ = 2.95 × 10^−4^ mNm^−1^.

Three needle diameters were selected for cavitation rheology measurements; 5 μm, 10 μm and 30 μm. At 37 °C, the critical pressure required to form a bubble (air into liquid) at the tip of needles with these internal diameters ranges from 4.6 to 28 kPa. Given that the elastic moduli of some cell types have been measured at ~100 Pa (Table 1), water was selected as the cavitation medium to reduce the air/water interfacial tension contribution to the critical pressure. The liquid used as the cavitation medium has minimal contact with the material (the only contact is at the interface between the liquid in the needle and the part of the cell in contact with the needle tip–and the contact only last for minutes, while the measurement is made). Cell culture medium is not used as the cavitation medium so as not to foul the pressure sensor (which is in direct contact with the liquid). The spheroids were however bathed in cell culture medium during the measurements to ensure that they remain alive. There is a minimal surface tension difference between water and the medium in which the cells are bathed. The values obtained from the cavitation rheology measurements are summarised in Table 2 and Fig. 3.

**Table 2:** Cavitation rheology of HEK 293 T/17 spheroids on day 7.

	Needle diameter		
	5 μm	10 μm	30 μm
Number of replicates	25	28	23
Minimum P_c_ (kPa)	0.047	0.014	0.013
Maximum P_c_ (kPa)	0.742	0.417	0.259
Relative frequency (0–0.15 kPa)	0.28	0.89	0.95
Relative frequency (0.15–0.25 kPa)	0.32	0.04	0.0
Relative frequency (0.25–0.50 kPa)	0.28	0.07	0.05
Relative frequency (0.5–0.80 kPa)	0.12	0.0	0.0

**Figure 4:**
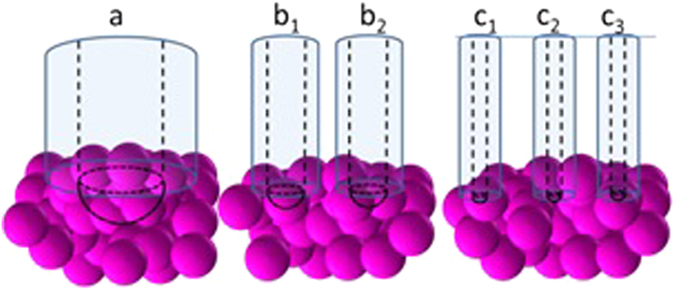
Schematic (to scale) of needles inserted into cell spheroids to illustrate the relative proportions of the needle diameters compared with the cell spheroid structures; diameter of needle a = 30 μm; b = 10 μm and c = 5 μm.

Before analysing the data, it is important to consider the dimensions of the needles relative to the components of the cell spheroid when considering what structures are being probed during cavitation measurements (Fig. 4). HEK293 cells have an average diameter of ~15 μm^63^. The 30 μm needle is much larger (2x) than the cell diameter, thus the needle is too large to probe the mechanics of a single cell and it is likely that any deformation that occurs upon bubble formation will be due to disruption of cell-cell contacts (rather than by deformation of individual cell structures). Hence, the material properties of cells around the local area of the needle tip and any associated adhesion molecules within the cell membranes should be reflected in the critical pressure measured at this needle diameter, Fig. 3a.

The 10 μm needle is comparable to the cell diameter, however, when the outside needle diameter is considered (exterior/interior diameter ratio = 1.33:1) this needle is also too large to penetrate a single cell (and keep it intact), Fig. 4b1. Again, for this needle size, it is the local extracellular environment and any cell-cell contacts that are the structures most likely to be probed by CR measurements at this length-scale, Fig. 4b2.

In principle, the 5 μm needle is sufficiently small to penetrate a single cell within the spheroid and enter the cytoplasm, even when the exterior diameter of the needle is accounted for. Therefore, for a 5μm needle, the material properties of the extracellular space and local cell-cell contacts, in addition to measurements inside a cell (albeit with lower frequency) are possible, Fig. 4c1–3.

For all three needle diameters the values determined for the critical pressure (Fig. 3) indicate that the deformation that does occur upon bubble formation is due to the disruption of the contacts between cells (rather than the individual structures within a single cell). The critical pressures observed in the vast majority of measurements, lie between 13 Pa and 500 Pa. Assuming that the deformation is elastic, averaging over all measurements a Young’s modulus of ≈20 Pa is estimated (from eq. 1). This is consistent with Young’s moduli measured using indentation and tensile deformations for soft biological tissues e.g. murine sarcoma at 700 Pa^23^, rat hippocampus, 201 Pa^33^ and colon cancer cells, 56–81 Pa^38^. Critical pressures exceeding 0.5 kPa (0.61, 0.66 and 0.74 kPa) occur infrequently and only for measurements made with a 5 μm needle (Fig. 3a). The elasticity of the cell cytoplasm ranges from 0.5 and 2.4 kPa^39, 64^ strongly suggesting that some of the measurements made with a 5 μm needle are indeed measuring elasticities for structures within a single cell.

### Interpretation of the data

The pressure required to form a cavity in a viscoelastic network is the sum of both the interfacial energy and the elastic restoring energy of the network. If we assume that the deformation in the cell bundle is elastic, i.e, that the critical pressure is proportional to 1/r, we can use eq. 1 to determine the interfacial tension, which is 2.95 × 10^–4 ^mNm^−1^ and the elastic modulus of the spheroid bundle is, ≈20 Pa, Fig. 3b.

Interfacial tension provides cells and tissues with apparent mechanical stiffness with which they resist mechanical stress. Interfacial tension values for individual cells (cortical tension) have been reported to range between 10^−3^ and 10^−5 ^mNm^−1^ ^17–21^ while tissue interfacial tensions have been measured at ~1.6 to 20 mNm^−1^ ^2, 22–24^. The interfacial tension that we have determined by CR, 2.95 × 10^−4 ^mNm^−1^, is within the range reported in the existing literature for cortical tension rather than tissue interfacial tension. The interfacial energy contributes minimally to the critical pressure at the length-scales probed (30 μm = 3.95 × 10^−5^ Pa, 10 μm = 1.18 × 10^−4^ Pa and 5 μm = 2.37 × 10^−4^ Pa). Therefore, at these length-scales the elastic modulus is the major contributor to the critical pressure.

Since the diameter of the needles, and hence the size of the bubble formed in the material at P_c_ is large relative to the size of the spheroid, it is prudent to consider if the “thickshell” limit is breached during the experiments. Using eq. 2, Solomon and co-workers showed that for large volumes [(R_i_ + H)/R_i_ ~ 20], the critical pressure reaches an asymptotic limit of ~5E/6 consistent with eq. 1. Whereas for finite volumes [(R_i_ + H)/R_i_ ~ 5] and [(R_i_ + H)/R_i_ ~ 1.5] the critical pressure was significantly lower, indicating that in this regime eq. 1 may not be valid. For the experiments described here, 15 < (R_i_ + H)/R_i_ < 91, suggesting that eq. 2 may better describe the relationship between *P*_*c*_ and *E*. Therefore we compared the values obtained using both eq. 1 and eq. 2 to determine if exceeding the “thickshell” limit had a significant impact on the values of *E* determined from the critical pressure. Since the interfacial tension contribution is small, i.e. 2γ/r ≈ 0 then eq. 1 becomes:

3 $${P}_{c}=5E/6$$

and eq. 2 becomes;

4 $${P}_{c}=\frac{5E}{6}[\begin{array}{c}\frac{6a}{5}{(\begin{array}{c}\frac{{R}_{i}+H}{{R}_{i}}\end{array})}^{b}+1\end{array}]$$

For eq. 4, the parenthetical contribution was calculated for each needle size and the local elastic moduli for each needle diameter was determined using both eqs 3 and 4 (Table 3) from the experimental data. There was almost no difference between the values for P_c_ determined from the experimental data using either eqs 3 and 4 (Table 3). Therefore, exceeding the “thickshell” limit in the experiments does not contribute strongly to the estimation of the elastic modulus.

**Table 3:** Calculations of local elastic moduli using eqs 3 and 4 where H = 200 μm (half the minimum spheroid diameter), *a* = −0.86 and *b* = −0.65^62^.

Needle diameter	Critical Pressure (kPa)	
	Equation 3	Equation 4
30 μm	39	32
10 μm	73	67
5 μm	227	220

Thus far, we have determined the critical pressure for the deformation of cell spheroids at 30 μm, 10 μm and 5 μm. If we assume that over this range of length-scales that the deformation is elastic, then the interfacial tension that we measure is consistent with the cortical tension for HEK cells. We have established that while the “thickshell” limit of CR is approached, that this does not significantly change the values for *E* or *γ* measured in the experiments. Therefore, we should be able to use this data to determine the contribution of cell-cell adhesion molecules on the surface of the cells within the spheroid to the elastic restoring energy.

Measuring the elastic modulus (for each needle radius) provides a means of determining the energy associated with the formation of the cavity at each needle diameter (length scale). These values may then be compared to the known adhesion properties of cell-cell contact molecules (mostly cadherins), that form attachments between the cells of the spheroid, predominantly via the hydrophobic effect^65^.

To determine the energy associated with the formation of a cavity at each needle radius, we consider the following: At the onset of cavity formation, the volume at the tip of the needle is that of a hemisphere with the same diameter as the internal diameter of the needle. The work of forming a bubble (*W*_*c*_) is related to the pressure of the medium (*P*_*m*_) and the volume of the bubble (*V*_*c*_) by;

5 $${W}_{c}={P}_{m}\times {V}_{c}$$

For each needle diameter, the energy associated with the formation of a cavity was determined. The cavity volumes were calculated to be 3.9 × 10^−17 ^m^3^, 2.6 × 10^−16 ^m^3^ and 7.1 × 10^−15 ^m^3^ for the needle diameters 5 μm, 10 μm and 30 μm, respectively. The corresponding work of cavity formation is therefore 7.4 × 10^−15 ^J, 1.9 × 10^−14 ^J and 2.8 × 10^−13 ^J, respectively (from eq. 5).

The contribution of cell-cell adhesion molecules to the elastic restoring energy must now be considered. Cadherins are an important family of proteins that mediate cell-cell adhesion. The adhesion process operates mainly via the hydrophobic effect and involves the insertion of an amino-terminal tryptophan residue from one strand into a hydrophobic pocket on the neighbouring strand^65^. To estimate the energy associated with cell-cell adhesion molecules, we make some assumptions. The energy associated with the formation of a cadherin-cadherin bond has been reported as 6.5 kcal mol^−1^ or 4.52 × 10^−20^ joules/bond (for N-cadherins)^66^. Both N- and E-cadherins are present on the cell surface, and E-cadherins associate with slightly lower energies (in the region of 5 kcal mol^−1^). However, HEK 293 cells have low levels of endogenous E-cadherin^67^, so for simplicity, we assume that the majority of the cadherins on the cell surface are N-cadherins (even if there is a 1:1 ratio of N- to E-cadherin, this would lead to an error in the region of 10% in our calculations). Total cadherin expression levels of have been reported to vary between 2.4 × 10^4^ and 15.8 × 10^4^ cadherins per cell for L929 cells^68^. Cadherin expression was determined from trypsinised, dissociated cells which have roughly spherical cell morphology with a diameter of 10 μm and therefore a cell surface area of 314 μm^2^. Again, here we are assuming the level of cadherin expression on the surface from a different cell line (since this data is not available for HEK 293). From the data for L929, we can estimate that there are between 7.6 × 10^1^ and 5.0 × 10^2^ cadherins per μm^2^. To relate this to HEK, we consider that dissociated HEK cells have a diameter of ~15 μm and a roughly spherical morphology with an estimated surface area of 706.5 μm^2^. Based on these two assumptions, we estimate that there are approximately 5.4 × 10^4^ cadherins/cell for HEK. Assuming an average bond energy of 6.5 kcal mol^−1^, this gives a total cadherin bond energy of 2.44 × 10^−15^ joules/cell (5.4 × 10^4^ cadherins/cell × 4.52 × 10^−20^ joules/cadherin).

The work of cavity formation (7.4 × 10^−15 ^J, 1.9 × 10^−14 ^J and 2.8 × 10^−13 ^J for needle diameters 5 μm, 10 μm and 30 μm, respectively), determined from our experimental data was divided by the cadherin bond energy that we calculate based on our assumptions of the cadherin bond energy and number of cadherins per cell (equal to 2.44 × 10^−15^ joules/cell). Therefore, the energy associated with the formation of the cavity (from experiments) was estimated to be equivalent to the disruption cadherin bonds on a surface area equivalent to ~3 cells for a 5 μm needle, ~8 cells for a 10 μm needle and ~115 cells for a 30 μm needle.

**Figure 5:**
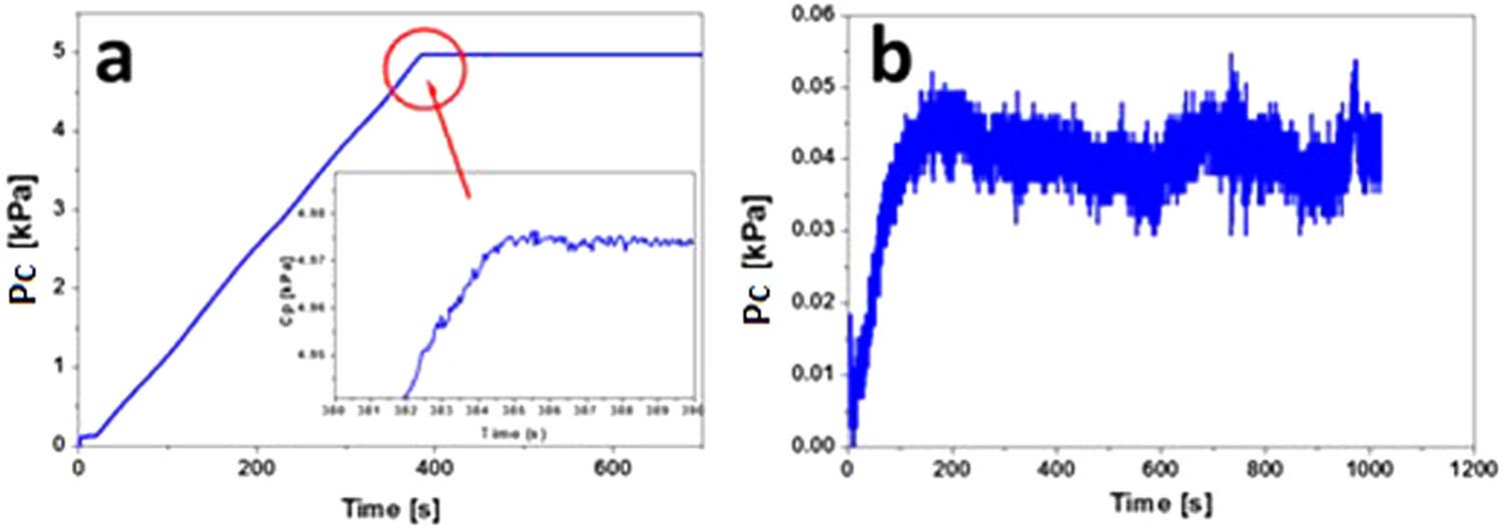
The cycling of pressure during a CR experiment; (**a**) using air as the cavitating medium into water, needle diameter = 30 μm, (**b**) using water as the cavitating medium into HEK.

A cascade of cell-cell dissociation events between the cells within the vicinity of the cavity must occur to facilitate the formation of a cavity. The area affected by such dissociations should increase with the volume of the cavity, which is scaled to the radius of the needle. The cavities formed by the 5 μm and the 10 μm needles occupy a small fraction of the volume of a HEK cell (x0.02 and x0.15, respectively). Although the cavity volume is smaller than a cell, the cell adhesion molecules on many cells around the cavitation zone are undoubtedly forced to disassociate to accommodate the formation of a bubble (Fig. 5). The cavity formed by a 30 μm needle has a volume four times that of a HEK cell. Therefore, we expect that cell adhesion molecules equivalent to those on many more cells must disassociate to accommodate such a relatively large bubble. Therefore, the total disrupted cell surface areas calculated, equivalent to 3 cells, 8 cells and 115 cells for 5, 10 and 30 μm needles, respectively, appear to be physically reasonable.

Given the very small volume change that occurs in the system upon cavitation using the needles sizes selected, as the needle radius decreases, the change in pressure associated with the formation of the bubble also decreases. This is shown for cavitation of an air bubble into water using a 30 µm needle (Fig. 5a). Indeed, when air was used to form a cavity in water, the bubble did not remain at the tip of the needle and continue to grow - rather the bubble detached from the needle tip and floated upwards. Thus, as the needle diameter decreases, the volume of the bubble prior to detaching, becomes increasingly small, minute compared to the total volume of the system (observed as small changes in pressure during a measurement). When the bubble detaches after cavitation, the pressure of the system is less than the critical pressure and surface tension restores the water surface at the needle tip. If the syringe pump was run continuously this results in a cycling of the system pressure over a narrow range of pressures. A similar effect was observed during the cavitation of the spheroids (Fig. 5b). If the syringe pump runs continuously, a cycling of the system pressure was also observed.

The surface tension contribution to the critical pressures (calculated from the bulk surface tension [for a 30 μm needle = 3.95 × 10^−5^ Pa; 10 μm = 1.18 × 10^−4^ Pa and 5 μm = 2.37 × 10^−4^ Pa]) are not sufficient to account for this effect, which must therefore originate in the elastic contribution to the critical pressure. If a deformation event resulting from fracture occurred, we would observe a decrease in the system pressure which would not recover. We do not observe this. A mechanism involving a rapid cycle of “unzipping” and “re-zipping” of the cadherin-cadherin contacts may explain this CR response. Upon the formation of the cavity the cadherin bonds dissociate. There is minimal surface tension between the water in the cavity and the culture medium in which the cells are bathed. The water can freely diffuse and the cadherin bonds are free to reform which restores the surface at the tip of the needle. Since bubble formation is rapid, less than 1 second, an elastic response is consistent with previous studies using fibroblasts^69^.

## Materials and Methods

### Cell culture

HEK293 cells were grown in a Memmert INCO 153 (Schwabach, Germany) CO_2_ incubator operated with 5% CO_2_, 90% relative humidity at 37 °C. Complete growth medium (CGM) was prepared from Hyclone® medium [Dulbecco’s Modified Eagles Medium/HIGH GLUCOSE, +4.00 mM L-Glutamine, +4,500 mg/l L-Glucose, -Sodium Pyruvate] (Thermo Scientific) which was supplemented with 10% heat inactivated bovine calf serum (Sigma-Aldrich) and 0.5% Penicillin (10,000 units) and streptomycin (10 mg/ml)(Sigma-Aldrich). Cell cultures were maintained by refreshing the CGM every 2–3 days as necessary. When the cultures reached 90% confluence, the cells were passaged.

The liquid overlay method was used to generate cell spheroids^70, 71^. A thin film of autoclaved 1% agarose was used to coat the bottom of a 96 well plate. 75 μl of a warm, sterile, agarose solution was dispensed into the interior wells of a 96-well plate to form a concave surface. The outermost wells were filled with 200 μl of sterile water. The plate was allowed to cool for one hour before use.

To prepare cells for transfer to the pre-prepared agarose plates, HEK293 cells at 90% confluence were rinsed with 1 × PBS after removal of CGM. The PBS was then replaced with 3 ml of trypsin-EDTA solution (Sigma-Aldrich). The flask was returned to the incubator for 10 minutes after which 10 ml of CGM was added and the cells were harvested by gently aspirating the medium to dislodge the cells from the wall of the flask. The cells were transferred to a centrifuge tube and spun at 125 × g for 10 minutes. The supernatant was discarded and the cells were re-suspended at a final concentration of 7.5 × 10^3 ^cells/ml. 200 μl of cell suspension was added to each agarose containing well, thus each well contained 1.5 × 10^3^ cells. The plates were placed in the incubator and the spheroid cultures were maintained by refreshing the CGM after 4 days. To avoid disruption to the spheroid, 150 μl of medium was gently aspirated from the wells and replaced with an equal volume of fresh medium which was gently pipetted along sidewall of the well. The spheroids were harvested on day 7.

### Light microscopy

Light microscopy was performed to monitor the growth of the spheroids. An Olympus CRX31 inverted microscope, equipped with a digital imaging system was used to view and record the images and all image analysis was done using ImageJ software^72^. A grid with known dimensions was used to measure spheroid sizes.

### Cavitation rheology (CR)

The cavitation rheology apparatus, built in-house has been previously described^73^. For the experiments performed in this paper the original PX26 series pressure sensor (Omega Engineering) was replaced by a High Accuracy Silicon Ceramic pressure sensor HSCDANT001PG3A3 (Honeywell). The needles used were µtip pre-pulled glass capillaries (World Precision Instruments; TIP05TW1F, TIP10TW1F and TIP30TW1F). A custom written programme recorded pressure at the needle tip during the experiments.

**Figure 6:**
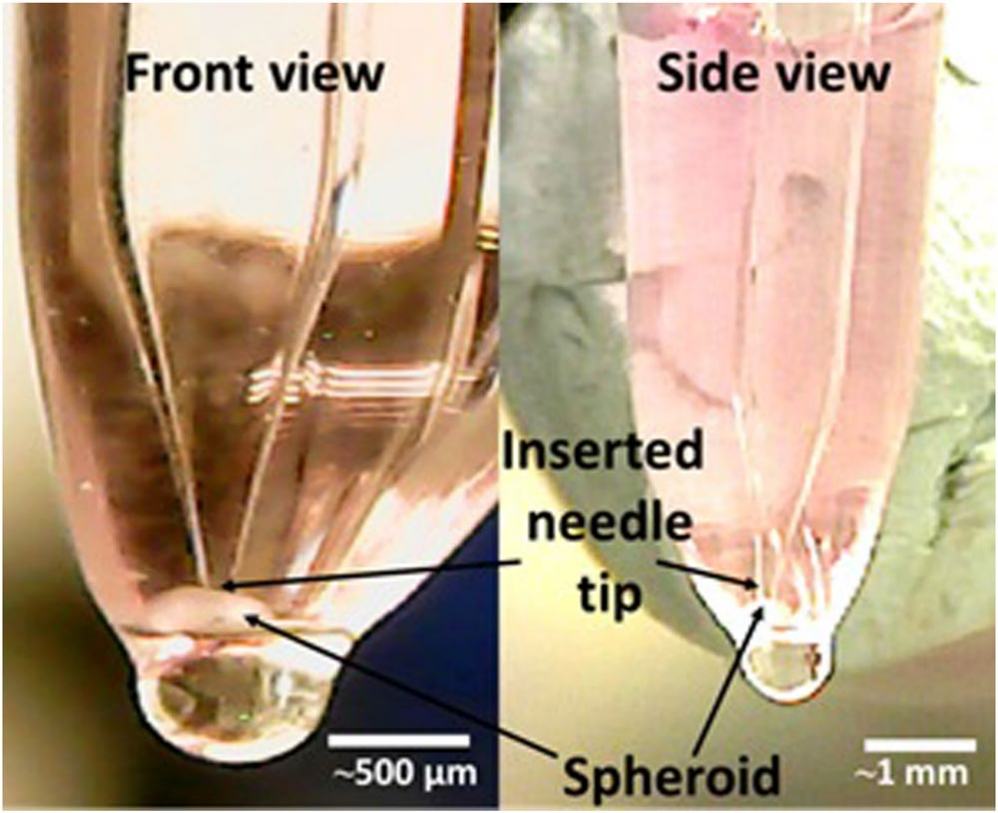
Insertion of a 30 μm needle into a spheroid–view from horizontal axes at two positions at a 90-degree separation.

For spheroid measurements, the spheroid was removed from the well of the 96 well plate by aspiration of the spheroid and surrounding medium using a P1000 Gilson pipette (Middleton, WI, USA), the tip being sufficiently large to allow the free passage of the spheroid. The spheroid was then transferred into a glass capillary (~1 mm diameter, closed at one end) along with a sufficient medium to bathe the spheroid. The needle in the CR instrument was attached to a custom made micro-manipulator. The needle was adjusted until the tip of the needle was inserted into the spheroid, at which time the cavitation rheology experiment began. The insertion process was monitored using cameras (with magnification) to ensure the correct placement of the needle tip (and to prevent penetration through the spheroid). Front and side views of the needle were monitored to ensure consistent and correct placement of the needle tip, Fig. 6. Cavitation rheology experiments were performed at a rate of 10 µl/min.

## Conclusions

Tissue and cell mechanics play an important role in the development of healthy organisms. Therefore, it is imperative to quantify the mechanical properties of biological material at this length scale. Here, the cavitation rheology technique has been extended to determine the elastic modulus and interfacial tensions in small volumes of biological soft matter. Using this novel approach, the interfacial tension at length-scales less than 30 μm is dominated by the cortical tension of the cells local to the needle point, for spheroids formed from HEK293 cells. Using the elastic modulus measured using cavitation rheology and previously reported values for the energy associated the formation of membrane bound adhesion molecules, the contribution of cell-cell adhesion to the elastic restoring energy was calculated. The mechanical response was related quantitatively to the disruption of cell-cell adhesion molecules during the formation of the cavity. A cascade of cadherin-cadherin dissociation events occurs to facilitate the formation of the cavity with a total disrupted cell surface areas equivalent to 3 cells, 8 cells and 115 cells for 5, 10 and 30 μm needles, respectively. While the extracellular matrix contributes to cell-cell interactions, we have not examined this in this study.

The deformation process involved appears to be predominantly elastic. When the syringe pump was run continuously a cycling of the system pressure over a narrow range of pressures was observed. The surface tension contribution to the critical pressures were not sufficient to account for this effect which must therefore originate in the reversible association of the cell-cell contacts. It is envisaged that the cadherin bonds undergo a rapid cycle of “unzipping” to accommodate the cavity and “re-zipping” to restore the surface at the needle tip.

The advancement of therapies to replace or regenerate damaged or injured tissues is crucial. Fundamental understanding of the physical characteristics of these tissues is paramount to the development of successful biomimetics with the desired functionality. The methodology and data presented herein provide a novel route to determine these properties and contributes towards a greater knowledge of physical principals involved in tissue mechanics.

## References

[1] Barone V, Heisenberg C-P (2012). Cell adhesion in embryo morphogenesis. Curr. Opin. Cell Biol..

[2] Manning ML, Foty RA, Steinberg MS, Schoetz E-M (2010). Coaction of intercellular adhesion and cortical tension specifies tissue surface tension. Proc. Natl. Acad. Sci. USA.

[3] Boivin, F. J., Sarin, S., Evans, J. C. & Bridgewater, D. The good and bad of β-catenin in kidney development and renal dysplasia. *Front. Cell Dev. Biol*., **3**, 81 1–10 (2015). 10.3389/fcell.2015.00081 PMC4686587 26734608

[4] Weiner OD, Marganski WA, Wu LF, Altschuler SJ, Kirschner MW (2007). An Actin-Based Wave Generator Organizes Cell Motility. PLoS Biol..

[5] Chang S-W, Buehler MJ (2014). Molecular biomechanics of collagen molecules. Mater. Today.

[6] Amiel D, Woo SL-Y, Harwood FL, Akeson WH (1982). The effect of immobilization on collagen turnover in connective tissue: A biochemical-biomechanical correlation. Acta Orthop..

[7] Schiele NR (2015). Actin cytoskeleton contributes to the elastic modulus of embryonic tendon during early development. J. Orthop. Res..

[8] Muiznieks LD, Keeley FW (2013). Molecular assembly and mechanical properties of the extracellular matrix: A fibrous protein perspective. Biochim. Biophys. Acta.

[9] Marturano JE, Arena JD, Schiller ZA, Georgakoudi I, Kuo CK (2013). Characterisation of mechanical and biochemical properties of developing embryonic tendon. Proc. Natl. Acad. Sci. USA.

[10] Foty RA, Steinberg MS (2005). The differential adhesion hypothesis: a direct evaluation. Dev. Biol..

[11] David, R. *et. al*. *Development*, **141**, 3672–3682 (2014). 10.1242/dev.104315 25249459

[12] Brasch J, Harrison OJ, Honig B, Shapiro L (2012). Thinking outside the cell: how cadherins drive adhesion. Trends Cell Biol..

[13] Steinberg MS (1963). Reconstruction of tissues by dissociated cells. Some morphogenetic tissue movements and the sorting out of embryonic cells may hav ea common explanation. Science.

[14] Harris AK (1976). Is cell sorting caused by differences in the work of intercellular adhesion? A critique of the Steinberg hypothesis. J. Theor. Biol..

[15] Graner F (1993). Can surface adhesion drive cell-rearrangement? Part I: Biological Cell-sorting. J. Theor. Biol..

[16] Brodland GW (2003). New information from cell aggregate compression tests and its implications for theories of cell sorting. Biorheology.

[17] Cartagena-Rivera AX, Logue JS, Waterman CM, Chadwick RS (2015). Actomysin Cortical Mechanical Properties in Nonadherent Cells Determined by Atomic Force Microscopy. Biophys. J..

[18] Hochmuth RM (2000). Micropipette aspiration of living cells. J. Biomech..

[19] Peukes J, Betz T (2014). Direct measurement of the cortical tension during the growth of membrane blebs. Biophys. J..

[20] Evans E, Yeung A (1989). Apparent viscosity and cortical tension of blood granulocytes determined by micropipet aspiration. Biophys. J..

[21] Chaigne A (2015). A narrow window of cortical tension guides asymmetic spindle positioning in the mouse oocyte. Nat. Commun..

[22] Foty RA, Pfleger CM, Forgacs G, Steinberg MS (1996). Surface tensions of embryonic tissues predict their mutual envelopment behavior. Development.

[23] Guevorkian K, Colbert M-J, Durth M, Dufour S, Brochard-Wyart F (2010). Aspiration of Biological Viscoelastic Drops. Phys. Rev. Lett..

[24] Mgharbel A, Delanoë-Ayari H, Rieu J-P (2009). Measuring accruately liquid and tissue surface tension with a compression plate tensiometer. HFSP J..

[25] Wyss HM (2015). Cell Mechanics: Combining Speed with Precision. Biophys J..

[26] Qi D (2015). Screening cell mechanotype by parallel microfiltration. Sci. Rep..

[27] Costa KD, Sim AJ, Yin FC-P (2006). Non-Hertzian approach to analyzing mechanical properties of endothelial cells probed by atomic force microscopy. J. Biomech. Eng..

[28] Schrot S, Weidenfeller C, Schäffer TE, Robenek H, Galla H-J (2005). Influence of hydrocortisone on the mechanical properties of the cerebral endothelium *in vitro*. Biophys. J..

[29] Sato H. *et. al*. *Colloids Surf. B. Biointerfaces*, **34**, 141–146 (2004). 10.1016/j.colsurfb.2003.12.013 15261083

[30] Mathur AB, Reichert WM, Truskey GA (2007). Flow and high affinity binding affect the elastic modulus of the nucleus, cell body and the stress fibers of endothelial cells. Ann. Biomed. Eng..

[31] Zeng D (2010). Young’s modulus of elasticity of Schlemm’s canal endothelial cells. Biomech. Model. Mechanobiol..

[32] Dokukin ME, Guz NV, Sokolov I (2013). Quantitative study of the elastic modulus of loosely attached cells in AFM indentation experiments. Biophys. J..

[33] Elkin BS, Azeloglu EU, Costa KD, Morrison III B (2007). Mechanical heterogeneity of the rat hipposcampus measured by afm indentation. J. Neurotrauma.

[34] Feneberg W, Aepfelbacher M, Sackmann E (2004). Microviscosity of the apical cell surface of human umbilical vein endothelial cells (HUVEC) within confluent monolayers. Biophys. J..

[35] Deguchi S, Maeda K, Ohashi T, Sato M (2005). Flow-induced hardening of endothelial nucleus as an intracellular stress-bearing organelle. J. Biomech..

[36] Khalilian M, Navidbakhsh M, Valojerdi MR, Chizari M, Yazdi PE (2010). Estimated Young’s modulus of zona pellucida by micropipette aspiration in combination with theoretical models of ovum. J. R. Soc. Interface.

[37] Tan SCW (2008). Viscoelastic behaviour of human mesenchymal stems cells. BMC Cell Biol..

[38] Pachenari M (2014). Mechanical properties of cancer cytoskeleton depend on actin filaments to microtubules content: Investigating different grades of colon cancer cell lines. J. Biomech..

[39] Caille N, Thoumine O, Tardy Y, Meister J-J (2002). Contribution of the nucleus ot the mechanical properties of endothelial cells. J. Biomech..

[40] Maeda E (2014). Significant increase in Young’s modulus of ATDC5 cells during chondrogeneic differentiation induced by PAMPS/PDMAAm double-network gel: comparison with induction by insulin. J. Biomech..

[41] Athanasiou KA, Eswaramoorthy R, Hadidi P, Hu JC (2013). Self-organization and the self-assembling process in tissue enginnering. Annu. Rev. Biomed. Eng..

[42] Stirbat TV (2013). Fine tuning of tissues’ viscosity and surface tension through contractility suggests a new role for α-catenin. PLoS One.

[43] Brodland GW, Veldhuis JH (2012). The mechanics of metastasis: insights froma computational model. PLoS One.

[44] Logue JS (2015). Erk regulation of actin capping and bundling of Eps8 promotes cortex tension and leader belb-based migration. Elife.

[45] Bose S, Das SK, Karp JM, Karnik R (2010). A semianalytical model to study the effect of cortical tension on cell rolling. Biophys. J..

[46] Bendas G, Borsig L (2012). Cancer cell adhesion and metastasis: selectins, integrins and the inhibitory potential of heparins. Int. J. Cell Biol..

[47] Cui J, Lee CH, Delbos A, McManus JJ, Crosby AJ (2011). Cavitation rheology of the eye lens. Soft Matter.

[48] Zimberlin JA, McManus JJ, Crosby AJ (2010). Cavitation rheology of the vitreous: mechanical properties of biological tissue. Soft Matter.

[49] Chin MS (2013). Cavitation rheology as a potential method for *in vivo* assessment of skin biomechanics. Plast. Reconstr. Surg..

[50] Sonmez M. *et. al*., H. Y. Ince, O. Yalcin, V. Ajdžanović, I. Spasojević, H. J. Meiselman and O. K. Baskurt, The effect of alcohols on red blood cell mechanical properties and membrane fluidity depends on their molecular size, *PLoS One*, **8**, e76579 (2013). 10.1371/journal.pone.0076579 PMC3781072 24086751

[51] Rodier F, Campisi J (2011). Four faces of cellular senescence. J. Cell Biol..

[52] Watters DA, Smith AN, Eastwood MA, Anderson KC, Elton RA (1985). Mechanical properties of the rat colon: the effect of age, sex and different conditions of storage. Q. J. Exp. Physiol..

[53] Jain RK, Au P, Tam J, Duda DG, Fukumura D (2005). Engineering vascularized tissue. Nat. Biotechnol..

[54] Rouwkema J, Rivron NC, van Blitterswijk CA (2008). Vascularization in tissue engineering. Trends Biotechnol..

[55] Shimizu S (1996). Induction of apoptosis as well as necrosis by hypoxia and predominant prevention of apoptosis by Bcl-2 and Bcl-XL. Cancer Res..

[56] Grimes DR, Kelly C, Bloch K, Partridge M (2014). A method for estimating the oxygen consumption rate in multicelluar tumor spheroids. J. R. Soc. Interface.

[57] Groebe K, Mueller-Klieser W (1996). On the relation between size of necrosis an diameter of tumor spheroids. Int. J. Radiat. Oncol. Biol. Phys..

[58] Wong C, Vosburgh E, Levine AJ, Cong L, Xu EY (2012). Human neuroendocrine tumor cell lines as a three-dimensional model for the study of human neuroendocrine tumor therapy. J. Vis. Exp..

[59] Alessandri K (2013). Cellular capsules as a tool for multicellular spheroid production and for investigating the mechanics of tumor progression *in vitro*. Proc. Natl. Acad. Sci. USA.

[60] Zimberlin JA, Sanabria-DeLong N, Tew GN, Crosby AJ (2007). Cavitation rheology for Soft Materials. Soft Matter.

[61] Gent AN (2005). Elastic Instabilities in rubber. Int. J. Non. Linear. Mech..

[62] Pavlovsky L, Ganesan M, Younger JG, Solomon MJ (2014). Elasticity of microscale volumes of viscoelastic soft matter by cavitation rheometry. Appl. Phys. Lett..

[63] Dietmair S (2012). A multi-omics analysis of recombinant protein production in Hek293 cells. PLoS One.

[64] Slomka N, Oomens CWJ, Gefen A (2011). Evaluating the effective shear modulus of the cytoplasm in cultured myoblasts subjected to compression using an inverse finite element method. J. Mech. Behav. Biomed. Mater..

[65] Maître J-L, Heisenberg C-P (2013). Three functions of cadherins in cell adhesion. Curr. Biol..

[66] Katsamba, P. *et. al*. *Proc. Natl. Acad. Sci. USA* **106**, 11594–11599 (2009).

[67] Hogan C (2004). Rap 1 Regulates the Formation of E-Cadherin-Based Cell-Cell Contacts. Mol. Cell. Biol..

[68] Duguay D, Foty RA, Steinberg MS (2003). Cadherin-mediated cell adhesion and tissue segregation: qualitative and quantitative determinants. Dev. Biol..

[69] Thoumine O, Ott A (1997). Time scale dependent viscoelastic and contractile regimes in fibroblasts probed by microplate manipulation. J. Cell Sci..

[70] Costa EC, Gaspar VM, Coutinho P, Correia IJ (2014). Optimization of Liquid Overlay Technique to Formulate Heterogeneic 3D Co-Culatures Models. Biotechnol. Bioeng..

[71] Carlsson, J. and Yuhas, J. M. Liquid-overlay culture of cellular spheroids, *Recent results cancer Res*. Fortschritte der Krebsforsch. Progrès dans les Rech. sur le cancer, **95**, 1–23 (1984). 10.1007/978-3-642-82340-4_1 6396753

[72] Schneider CA, Rasband WS, Eliceiri KW (2012). NIH Image to ImageJ: 25 years of image analysis. Nat. Method.

[73] Blumlein A, McManus JJ (2015). Bigel formation via spinodal decomposition of unfolded protein. J. Mater. Chem. B.

